# Reversal of perturbed DNA damage response and HIV latency by a histone methyltransferase inhibitor in virally suppressed individuals living with HIV

**DOI:** 10.3389/fragi.2025.1658506

**Published:** 2025-09-09

**Authors:** Pragati Chavan, Sathi Koar, Rohini Bingewar, Anuja Sonawane, Jyoti Sawant, Pallavi Shidhaye, Amrita Rao, Rajani Bagul, Ujjwala Ghule, Sunita Kumbhar, Manisha Ghate, Ashwini Shete

**Affiliations:** ICMR-National Institute of Translational Virology and AIDS Research (formerly ICMR-National AIDS Research Institute), Pune, India

**Keywords:** PLHIV, global DNA hypomethylation, 8-OHdG, prelamin A, oxidative stress

## Abstract

**Background:**

Oxidative stress contributes to DNA damage, further leading to cellular senescence. HIV infection increases oxidative stress and interferes in DNA damage response. Hence, a study was conducted to assess the extent of DNA damage by evaluating global methylation, 8-hydroxy-2′-deoxyguanosine (8-OHdG), and prelamin A levels in people living with HIV (PLHIV), representing alterations at the genetic, epigenetic, and structural levels. We also investigated the effect of methylation modulators on these markers and HIV latency reversal.

**Methods:**

Middle-aged virally suppressed PLHIV on long-term antiretroviral therapy (ART) and non-infected controls were enrolled for the study. The levels of 5-methylcytosine in blood and plasma 8-OHdG were assessed using commercially available colorimetric and ELISA kits, respectively. Reactive oxygen species (ROS) and prelamin A expression in T cells were assessed by flow cytometry. The levels of DNA damage markers were analyzed for their correlation with immunosenescent and inflammatory markers. Samples were treated with RG108 and chaetocin to assess their effect on these markers and HIV reactivation. HIV reactivation was assessed by the expression of intracellular P24 by flow cytometry and gag copies by real-time PCR.

**Results:**

PLHIV had significantly lower global DNA methylation levels and higher 8-OHdG and prelamin A levels than their age-matched HIV-uninfected controls. The ROS levels did not differ significantly among them. Global methylation and prelamin A levels correlated with immunosenescent T cells. The 8-OHdG levels correlated positively with angiotensin-II levels and negatively with proinflammatory cytokines. Treatment with chaetocin increased the global methylation levels and diminished prelamin A accumulation in CD4^+^ T cells in PLHIV. P24-expressing CD4+T cells increased significantly after chaetocin treatment, indicating HIV latency reversal.

**Conclusion:**

PLHIV on ART had higher DNA damage-related markers despite having comparable ROS levels than their age-matched controls. The immunotherapeutic potential of chaetocin for reversing premature aging as well as HIV latency needs to be explored further.

## 1 Introduction

People living with HIV (PLHIV) are at a higher risk of premature aging, with their biological age exceeding that of their HIV-negative peers by almost 5 years (Horvath and Levine, 2015). The higher risk of accelerated aging is in spite of the successful antiretroviral treatment (Sehl et al., 2020) that causes viral suppression and reduces the risk of AIDS-related events. Chronic persistent inflammation is the major factor driving age acceleration in these individuals (Nasi et al., 2017). Chronic inflammation is responsible for oxidative stress in PLHIV, which might induce DNA damage at epigenetic and genetic levels (Zhu et al., 2021).

It has now been established that epigenetic factors serve as the major determinant of aging by regulating the expressions of various genes. One of the epigenetic changes associated with the aging process is global DNA hypomethylation. Methylation of repetitive sequences including Alu and long-interspersed nuclear element (LINE-1) is associated with the total genomic methylation content. Loss of methylation of such sequences results in DNA hypomethylation (Erichsen et al., 2018). Oxidative stress causes global hypomethylation (Furlan et al., 2017). Oxidative stress induces the expression of DNA methyltransferases (DNMTs) or causes DNA damage, altering its structure, which further recruits DNMTs at the site of the damage, thus increasing the interaction between DNA and DNMTs (O'Hagan et al., 2011). Oxidative stress also causes histone modifications by altering their acetylation or methylation status. Global hypomethylation is considered to be the hallmark of carcinogenesis and is also associated with various age-related disorders (Tsang et al., 2016).

Apart from inducing epigenetic changes, oxidative stress is also known to induce changes at the genetic levels. Guanine gets oxidized to 8-hydroxy-2′-deoxyguanosine (8-OHdG) under oxidative stress conditions. Since 8-OHdG can pair with adenine and cytosine, transversion mutations may occur during cell replication (Suzuki and Kamiya, 2017). 8-OHdG is a biomarker of generalized, cellular oxidative stress and might also be a risk factor for cancer, atherosclerosis, and diabetes (Wu et al., 2004). Angiotensin-II treatment has been shown to increase the levels of 8-OHdG by acting through the angiotensin-II/AT_1_R pathway by increasing free radical production (Uemura et al., 2008), and it is also shown to accelerate cellular senescence (Herbert et al., 2008). On the contrary, 8-OHdG is also known to initiate a regulatory feedback mechanism by serving as a potent antioxidant and anti-inflammatory agent by downregulating the expressions of Rac-linked NADPH oxidase, iNOS, and Cox2 (Ock et al., 2012).

Similarly, oxidative stress also results in the accumulation of prelamin A, even in the normal aging process. Prelamin A is a precursor protein that is processed into mature lamin A, a structural component of the nuclear lamina. This accumulation is partly caused by downregulation of the expression of the processing enzyme FACE1 (Ragnauth et al., 2010), which is responsible for the final cleavage of prelamin A, transforming it into mature lamin A. Structural alterations in the nucleus due to prelamin A accumulation result in loss of peripheral heterochromatin, affecting the regulation of gene expression and DNA replication (Goldman et al., 2004). Thus, prelamin A accumulation hinders the DNA damage response, leading to genomic instability and premature aging (Ragnauth et al., 2010). It contributes to the aging process in physiological conditions and in PLHIV apart from its demonstrated causative role in Hutchinson–Gilford progeria syndrome (Lefevre et al., 2010; Scaffidi and Misteli, 2006).

The aging process is known to be linked with DNA methylation, which in turn is mediated by DNMTs. Modulation of DNMT activity has been shown to influence the aging process and longevity (Johnson et al., 2012). Similar to DNMTs, histone methyltransferases (HMTs) also mediate the aging process by regulating the expression of various genes. Apart from mediating the aging process, DNA and histone methylation also contribute to maintenance of HIV latency, which may be reversed using the DNMT and HMT inhibitors (Battistini and Sgarbanti, 2014). Hence, we conducted a study to determine if PLHIV have altered DNA damage response processes than their HIV-negative peers by assessing the epigenetic, genetic, and structural factors associated with DNA damage. We also determined the effect of methylation modulators on these factors, which might contribute to reversal of aging and HIV latency in PLHIV. We evaluated the roles of RG108, a non-nucleoside DNMT inhibitor, and chaetocin, an HMT inhibitor, on aging and latency reversal in our study. Both these modulators have previously demonstrated their role in slowing the progression of cardiovascular diseases (Shi et al., 2022); however, their role in HIV latency reversal is unexplored.

## 2 Methodology

### 2.1 Study population

The study had a case–control design. The study participants belonged to the age group of 40–55 years and were enrolled from the clinics and free ART centers affiliated with the institute, as described previously (Shete et al., 2024). The study participants were asymptomatic and free of any active acute or chronic co-infections. Potential study participants were contacted for voluntary participation and blood donations as per the study procedure requirements. They were enrolled after obtaining written informed consents from them. PLHIV successfully responding to first-line ART for more than 5 years, as indicated by an undetectable viral load at the time of enrollment, were enrolled as “cases” (n = 70). HIV-negative individuals matched with the cases for age and sex were enrolled as “controls” (n = 68). Whole-blood samples, plasma, and peripheral blood mononuclear cells (PBMCs) were cryopreserved for subsequent analysis.

### 2.2 Flow cytometry for detection of reactive oxygen species and prelamin A expression

Reactive oxygen species (ROS) production in PBMCs was detected using the dye 2′,7′-dichlorofluorescein diacetate (DCFDA), which gets converted to the highly fluorescent 2′,7′-dichlorofluorescein (DCF) upon oxidation, indicating the intracellular level of ROS. PBMCs of the study participants were revived and stained with the DCFDA cellular ROS assay kit (Abcam, United Kingdom) according to the manufacturer’s instructions for assessing ROS production. The expression of markers of CD4^+^ and CD8^+^ T-cell activation along with markers of T-cell senescence were determined by flow cytometry, as described previously (Shete et al., 2024).

Prelamin A accumulation was assessed by intracellular flow cytometry using anti-prelamin A (clone PL-1C7) antibody. PBMCs of the study participants were fixed, permeabilized, and blocked for 3 h at 4 °C with intermittent washing, as described elsewhere (Casasola et al., 2016). The cells were incubated with the primary antibody in the blocking buffer for 12 h at 4 °C and then with the secondary antibody and other surface and intracellular antibodies (CD3, CD8, and P24) for 30 min at 4 °C. Cells were resuspended in the sheath after washing and analyzed by flow cytometry using BD-FACS Fusion (BD biosciences, United States). CD4 T cells were identified as CD3^+^ and CD8-negative cells as HIV is known to cause CD4 downregulation (Richard et al., 2024). The list of the markers and their respective fluorochromes is given in Table 1.

**TABLE 1:** List of antibodies used in flow cytometry assays.

Assay tubes	Name of the marker	Fluorochrome
Tube 1: Prelamin a assay	Viability marker	BV510
	CD3	PECF594
	CD8	PE
	P24	FITC
	Prelamin A	BV421
Tube 2: DCFDA assay	Viability marker	BV510
	Anti-CD3	APCH7
	Anti-CD8	BUV737
	Anti-CD45	BV786
	Anti-CCR7	BUV395
	Anti-CD25	APC
	Anti-HLA-DR	PerCP-Cy5.5
	Anti-CD38	PE
	Anti-CD57	PECF594
	Anti-CD28	PECy7
	ROS	DCFDA

### 2.3 Detection of global DNA methylation

Global DNA methylation levels were determined by measuring 5-methylcytosine (5MedCyd) using a commercially available colorimetric assay kit (Abcam, United Kingdom). DNA was extracted from whole blood using a DNAeasy mini kit and used for the detection of global methylation following the manufacturer’s instructions. Optical density (ODs) values were determined using a microplate reader at 450 nm within 15 min. The percentage of methylated DNA was calculated using the following formula:

$$5-\text{mC}\%=\frac{\text{Sample}\;\text{OD}-\text{Negative}\;\text{Control}\;\text{OD}}{\text{Slope}\times \text{amount}\;\text{of}\;\text{input}\;\text{sample}\;\text{DNA}\;\text{in}\;\text{ng}}*100\%.$$

### 2.4 Estimation of 8-OHdG and angiotensin-II levels by ELISA

The 8-OHdG and angiotensin-II levels were estimated using commercially available ELISA kits (Elabscience, Texas, United States) in accordance with the manufacturers’ instructions. The sensitivity of the 8-OHdG ELISA kit was 0.94 ng/mL, and that of the angiotensin-II ELISA kit was 18.75 pg/mL.

### 2.5 Estimation of proinflammatory cytokines by multiplex luminex assay

Plasma samples of the study participants were processed for the detection of proinflammatory cytokine profile using the luminex assay (Bio-Rad Laboratories, United States) according to the manufacturer’s instructions. The cytokines assessed were interferon (IFN)-γ; interleukin (IL)-1b, IL-2, IL-6, IL-8, IL-12, IL-18; and tumor necrosis factor (TNF)-α. The plates were read using the Bio-plex 200 system and analyzed using Bio-Plex Manager software (Bio-Rad Laboratories, United States).

### 2.6 Experiments with methylation modulators

Methylation modulators such as RG108 and chaetocin were obtained from a commercial source (Sigma-Aldrich, United States). Both were first evaluated for their toxicity and dose in whole blood using four concentrations, namely, 5 µM, 10 µM, 15 µM, and 30 µM for RG108 and 50 nM, 100 nM, 200 nM, and 400 nM for chaetocin. Cytotoxicity was determined by live–dead staining using BV421 dye (Thermo Fisher Scientific, United States) and by flow cytometry using BD-FACS Fusion (BD biosciences, United States). The percentage viability after the treatment with all four concentrations was more than 90% (data not shown). Hence, the highest concentrations for both the molecules were chosen for further experiment.

### 2.7 HIV reactivation after treatment with methylation modulators

PBMCs isolated from PLHIV (n = 8) were treated separately with RG108 (30 µM) and chaetocin (400 nM) for 24 h in a humidified 5% incubator at 37 °C. HIV reactivation was determined by assessing the intracellular P24 expression in CD4^+^ T cells by flow cytometry using BD-FACS Fusion (BD biosciences, United States).

Furthermore, CD4^+^ T cells enriched from the blood of PLHIV cases (n = 18) using RosetteSep^tm^ Human T-cell enrichment cocktail (STEMCELL Technologies, Canada) were incubated for 24 h with RG108 (30 uM) and chaetocin (400 nM) at 37 °C for determining HIV-1 gag expression by real-time PCR assay, as described previously (Shete et al., 2022).

### 2.8 Prelamin A expression after treatment with methylation modulators

The effect of methylation modulators on prelamin A accumulation in PBMCs (n = 8) was assessed by the flow cytometry experiments described in the previous section. PBMCs of the study participants were incubated with RG108 (30 µM) and chaetocin (400 nM) separately in a 48-well plate in a humidified 5% CO_2_ incubator at 37 °C for 48 h and processed for prelamin A staining. Treated cells were considered the positive control, and untreated cells were considered the negative control.

### 2.9 Global DNA methylation levels after treatment with methylation modulators

The effect of methylation modulators on global DNA methylation levels in whole-blood cell DNA (n = 16) was checked by using the commercially available colorimetric assay kit described in the previous section. Whole-blood samples of the study participants were incubated separately with RG108 (30 µM) and chaetocin (400 nM) in a humidified 5% CO_2_ incubator at 37 °C for 24 h and processed for determining the global DNA methylation levels after extracting DNA.

### 2.10 Statistical analysis

GraphPad Prism software version 9 was used for performing data analysis and plotting graphs. Nonparametric tests such as Mann–Whitney U and Wilcoxon signed-rank tests were used for comparing unpaired and paired data from the study participants, respectively. Spearman’s rank test was used for the correlation analyses between various parameters. P-values below 0.05 were considered significant.

## 3 Results

### 3.1 Study participants

Baseline characteristics of the cases and controls enrolled in the study were published previously (Shete et al., 2024). Briefly, cases and controls in our study did not differ in terms of age (median: 47 years and interquartile range: 42 years–52 years for controls and median: 47 years and interquartile range: 42 years–51 years for cases) and sex ratio (male: female ratio: 31:37 for controls and 36:34 for cases). However, the cases had significantly lower CD4 counts than the controls (p < 0.0001).

### 3.2 Comparison of the levels of ROS and DNA damage-related markers in cases and controls

**FIGURE 1:**
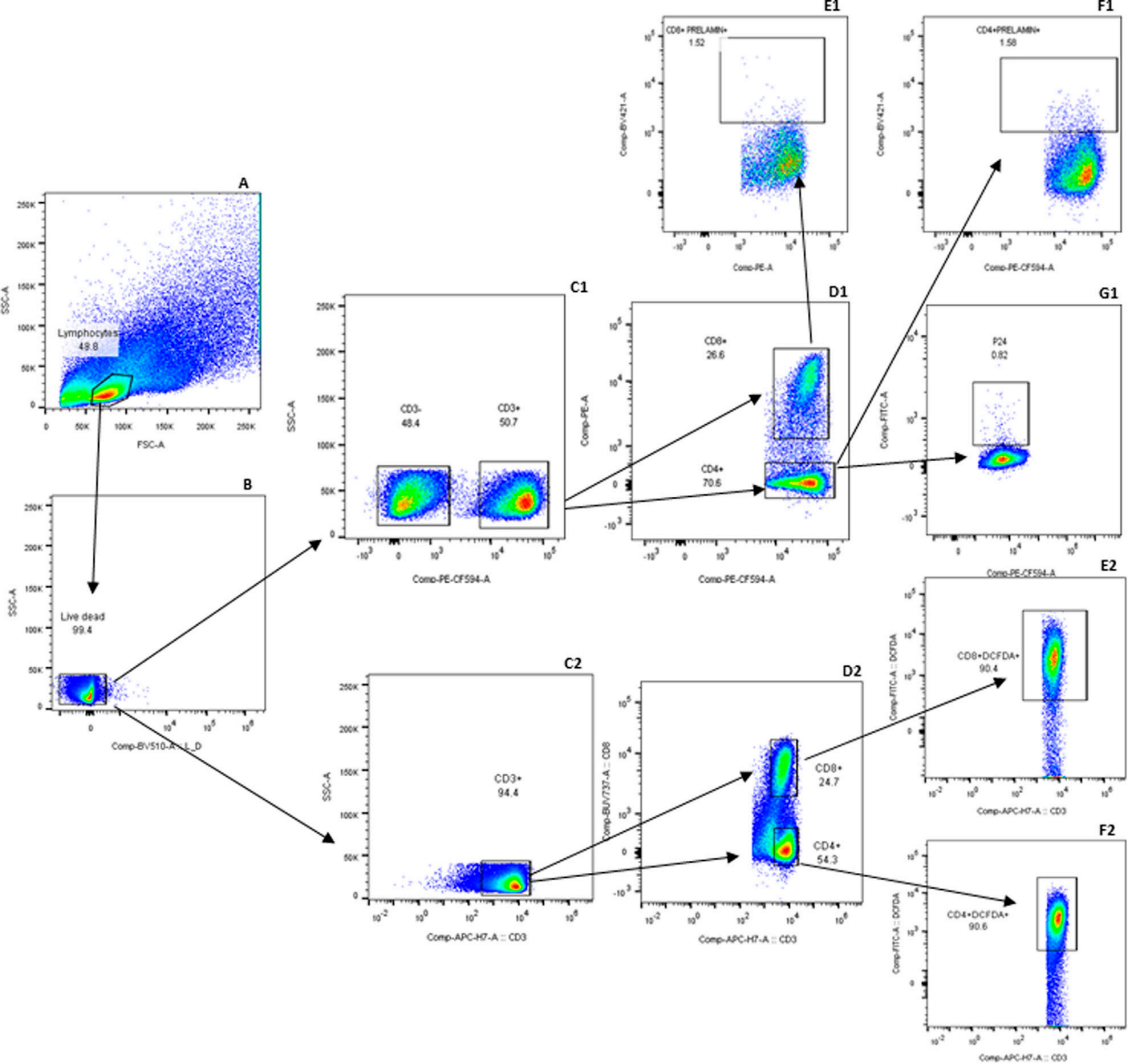
Gating strategy for detecting prelamin A and DCFDA in T-cell subsets: expressions of prelamin A and DCFDA were assessed in two separate tubes. The gating strategy in the first tube is labeled with a series of letters followed by 1, and that for the second tube is 2. Lymphocytes were first identified based on forward and side scatter **(A)** and then gated for viable cells **(B)**. Viable cells were further gated to identify T cells based on CD3 expression (C1 and C2). Within the CD3^+^ population, CD4^+^ and CD8^+^ T cells were identified (D1 and D2). CD8^+^ T cells were further analyzed for prelamin A (E1) and DCFDA (E2) expression. Similarly, CD4^+^ T cells were assessed for prelamin A (F1), P24 (G1), and DCFDA (F2) expressions. FMx controls stained with viability and surface markers for T cells were used to distinguish populations shown in the gating strategy. Gated CD3, CD4, and CD8 T cells, which were not stained for other markers, were further used for gating markers included in the tubes.

**FIGURE 2:**
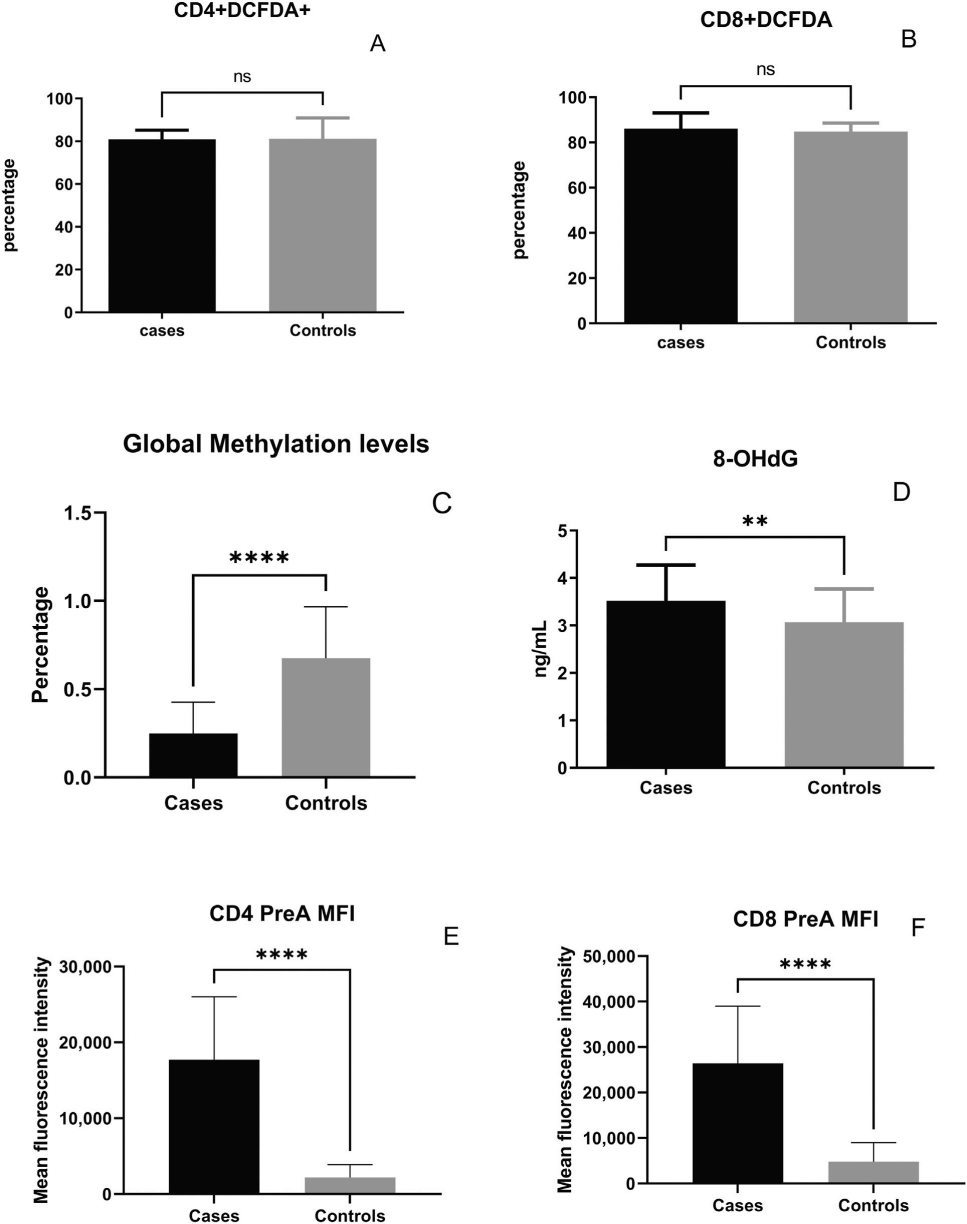
Levels of reactive oxygen species and DNA damage-related markers in the cases and controls: percentage of DCFDA-expressing CD4^+^ T cells **(A)**, percentage of DCFDA-expressing CD8^+^ T cells **(B)**, percentage of global methylation **(C)**, plasma 8-OHdG levels **(D)**, and the mean fluorescence intensity (MFI) of prelamin A expression in CD4^+^ **(E)** and CD8^+^ T-cells **(F)** are shown on the y-axis. Bars in the figure represent the medians, and error bars indicate interquartile ranges for the values. P-values of the unpaired data were calculated using Mann–Whitney U test. (NS- not significant p-value, **: p < 0.01, and ****: p < 0.0001).

Prelamin A accumulation and DCFDA expression indicating ROS levels among different populations of T cells was assessed by staining the cells in two different tubes for flow cytometry-based assay. The gating strategy used for flow cytometry analysis is reported previously (Shete et al., 2024), and the newer markers included in the analysis were gated according to the strategy shown in Figure 1. Comparison of the levels of ROS and DNA damage-related markers such as global DNA methylation levels, 8-OHdG levels, and prelamin A expression in T cells in the cases and controls is shown in Figure 2. No significant difference in ROS levels was detected in CD4^+^ and CD8^+^ T-cells between the cases and controls when assessed by a DCFDA cellular ROS assay kit. Global DNA methylation levels in blood cells were significantly lower in PLHIV than in their age-matched HIV-negative controls (p < 0.0001), whereas 8-OHdG levels were significantly higher in PLHIV than in the controls (p = 0.0017). Prelamin A expression in CD4^+^ and CD8^+^ T cells (p < 0.0001 for both) was also significantly higher in cases than in controls.

### 3.3 Correlation of global DNA hypomethylation levels with the markers of immunosenescence

**FIGURE 3:**
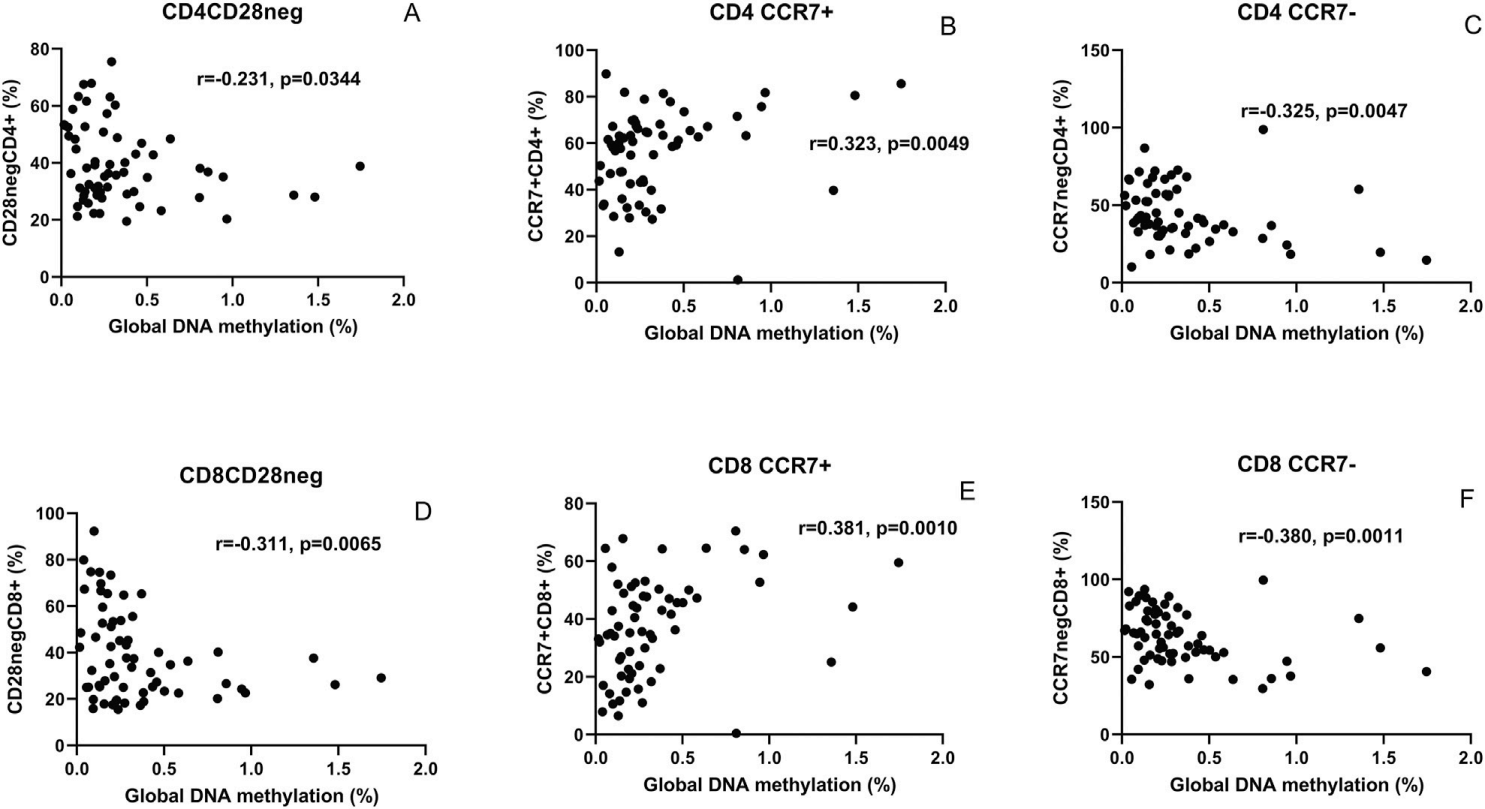
Correlation analysis of global DNA hypomethylation levels with markers of immunosenescence: scatter plot shows correlation between the global methylation levels (x-axis) and CD4^+^ and CD8^+^ T cells lacking CD28 expression (**(A,D)**, respectively), expressing CCR7 (**(B,E)**, respectively), and lacking CCR7 expression (**(C,F)**, respectively) plotted on the y-axis. The correlation coefficient (r) and (p) values as assessed by Spearman test are mentioned in the plots.

Correlations of the global DNA hypomethylation levels with immunosenescent T cells identified based on the absence of CD28 and presence of CCR7 expression are shown in Figure 3. Global DNA methylation levels correlated negatively with CD28-negative CD4^+^ (r = −0.231, p = 0.034, one-tailed) and CD8^+^ T cells (r = −0.311, p = 0.006). The levels correlated positively with CCR7-expressing CD4^+^ (r = 0.323, p = 0.0049) and CD8^+^ T cells (r = 0.381, p = 0.001). Conversely, a negative correlation of the levels was observed with CD4^+^ (r = −0.325, p = 0.0047) and CD8^+^ T cells (r = −0.380, p = 0.0011) lacking CCR7 expression.

### 3.4 Correlation of 8-OHdG levels with angiotensin-II and proinflammatory cytokines

**FIGURE 4:**
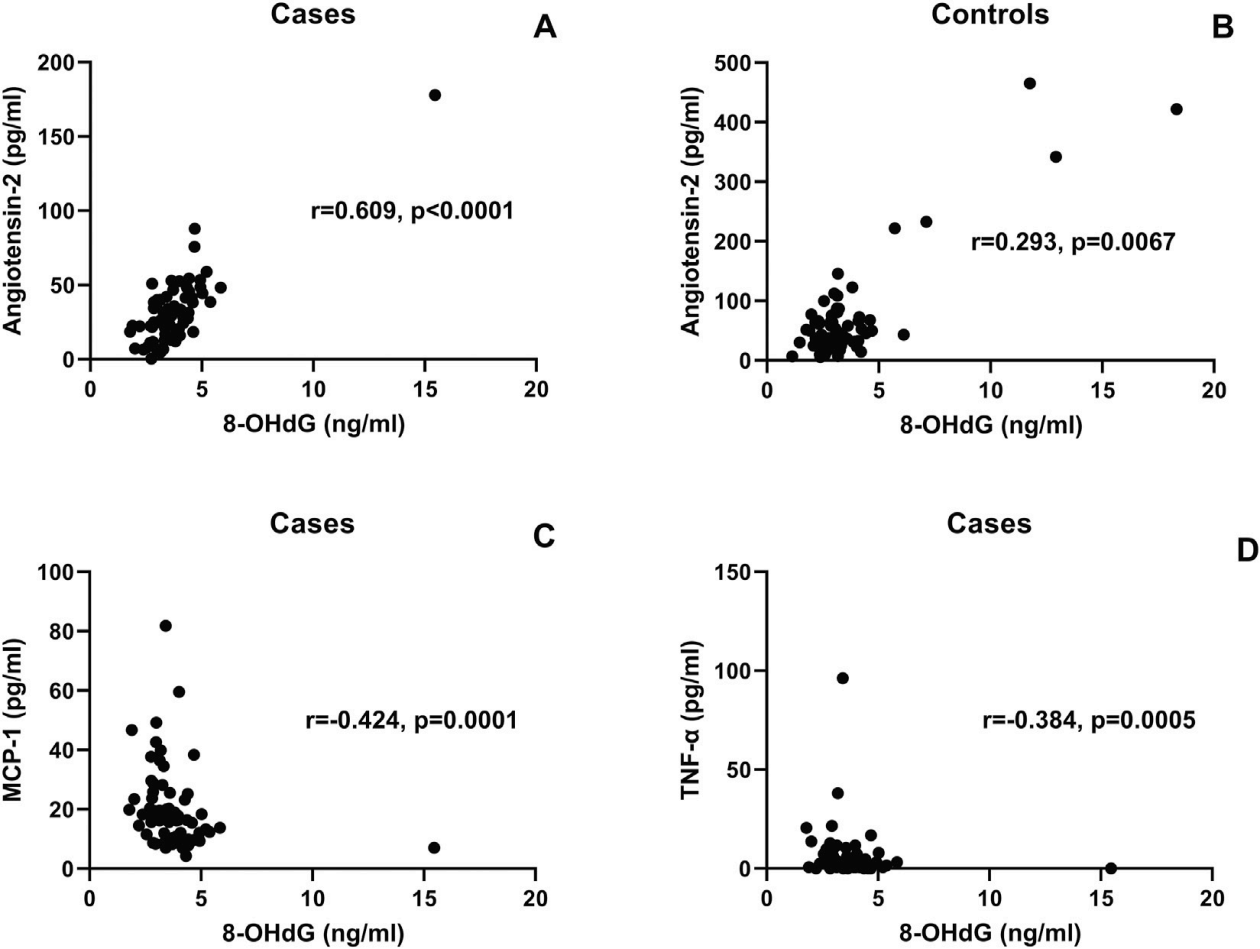
Correlation of the 8-OHdG levels with angiotensin-II and proinflammatory cytokines: the scatter plot shows the correlation between plasma 8-OHdG levels (x-axis) and plasma angiotensin-II (y-axis) in the cases **(A)** and controls **(B)**. Correlation between plasma 8-OHdG levels (x-axis) and MCP-1 and the TNF-α levels plotted on the y-axis is shown in scatter plots **(C,D)**, respectively. The correlation coefficient (r) and (p) values, as assessed by Spearman test, are mentioned in the plots.

Correlations of the 8-OHdG levels with angiotensin-II and proinflammatory cytokines are shown in Figure 4. 8-OHdG levels were found to correlate with angiotensin-II levels in the cases (r = 0.609, p < 0.0001) and controls (r = 293, p = 0.016). Surprisingly, 8-OHdG levels correlated negatively with proinflammatory cytokines such as MCP-1 (r = −424, p < 0.0001) and TNF-α (r = −384, p = 0.0005).

### 3.5 Correlation of prelamin A expression with senescent and activated CD4^+^ T-cells

**FIGURE 5:**
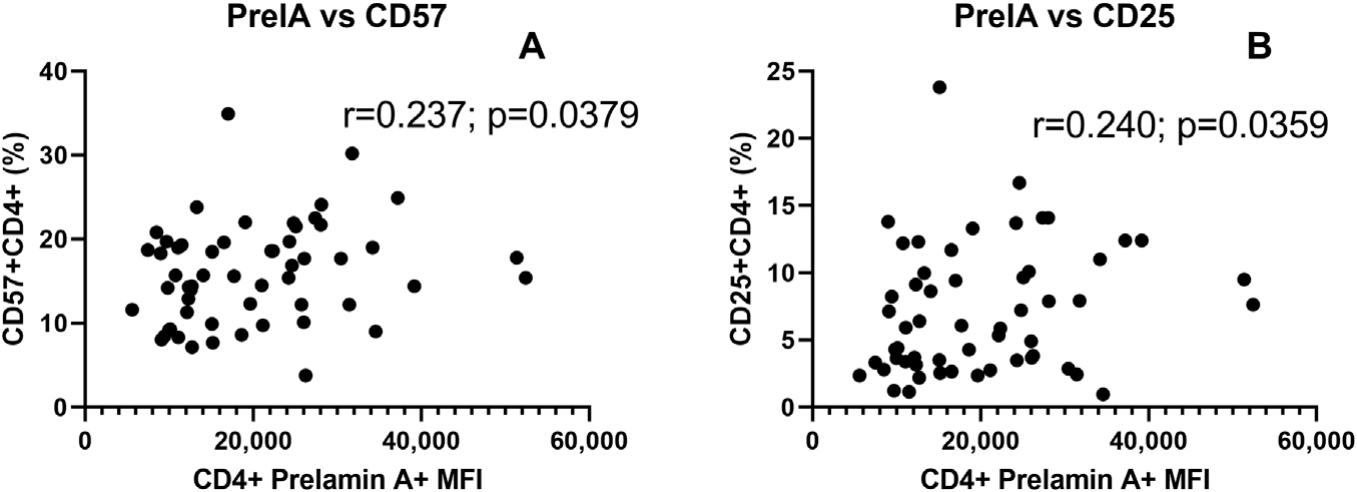
Correlation of prelamin A expression with senescent and activated CD4+ T-cells: the scatter plot shows the correlation between MFI of prelamin A expression in CD4^+^ T cells (x-axis) and the frequency of CD4^+^ T cells expressing senescence marker CD57^+^ **(A)** and immune activation marker CD25 **(B)** plotted on the y-axis. The correlation coefficient (r) and (p) values as assessed by Spearman test are mentioned in the plots.

Correlations of prelamin A expression with CD4^+^ T cells expressing CD57 and CD25 representing senescent and activated T cells are shown in Figure 5. The levels of prelamin A expression in CD4^+^ T cells were found to correlate with the frequency of CD57^+^ CD4^+^ (r = 0.237, p = 0.0379, one-tailed) and CD25^+^ CD4^+^ (r = 240, p = 0.0359, one-tailed) T cells.

### 3.6 The effect of chaetocin and RG108 treatment on the levels global DNA methylation and prelamin A expression

**FIGURE 6:**
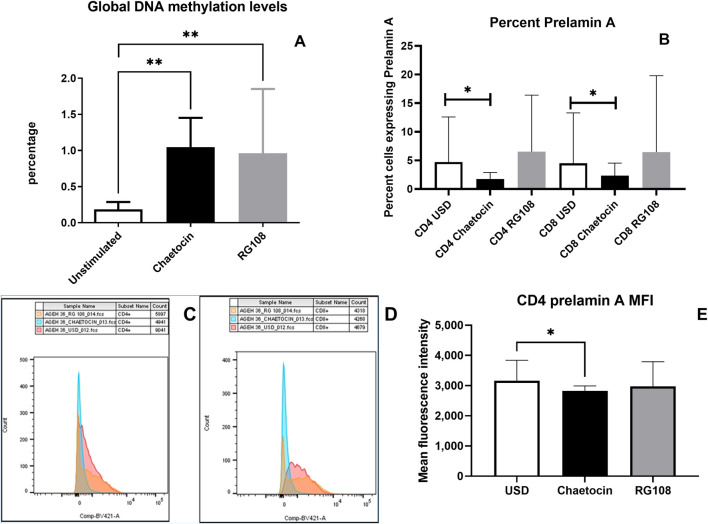
Effect of chaetocin and RG108 treatment on the levels of global DNA methylation and prelamin A expression: **(A)** global methylation levels in the study participants before and after the treatment with chaetocin and RG108 are shown in the figure. **(B)** Bar graph showing the percent of CD4^+^ and CD8^+^ T cells of PLHIV expressing prelamin A before and after the treatment with chaetocin and RG108. **(C, D)** Flow cytometry histogram showing the MFI of prelamin A expression in unstimulated (red), chaetocin-treated (blue), and RG108-treated (orange) CD4^+^ and CD8^+^ T-cells. **(E)** MFI of prelamin A expression in CD4^+^ T cells of PLHIV before and after treatment with chaetocin and RG108. (p values <0.05 are indicated as *, and those <0.01 are indicated as **).

The effect of chaetocin and RG108 treatment on the levels of global DNA methylation and prelamin A expression is shown in Figure 6. Treatment with chaetocin and RG108 caused a significant increase in global methylation levels (p = 0.0052 and p = 0.0092, respectively). Conversely, treatment with chaetocin caused a significant decrease in the frequency of CD4^+^ and CD8^+^ T cells expressing prelamin A (p = 0.0293 and p = 0.0273, respectively, both one-tailed). The MFI of prelamin A expression in CD4^+^ T cells also decreased significantly after chaetocin treatment (p = 0.0273, one-tailed). Representative overlay histograms of prelamin A expression in unstimulated, chaetocin- and RG108-treated CD4 T-cells are shown in Figures 6C, D. However, no such decrease in prelamin A expression in CD4^+^ or CD8^+^ T cells in terms of frequency and MFI was observed after RG108 treatment.

### 3.7 The effect of chaetocin and RG108 treatment on HIV latency reversal

**FIGURE 7:**
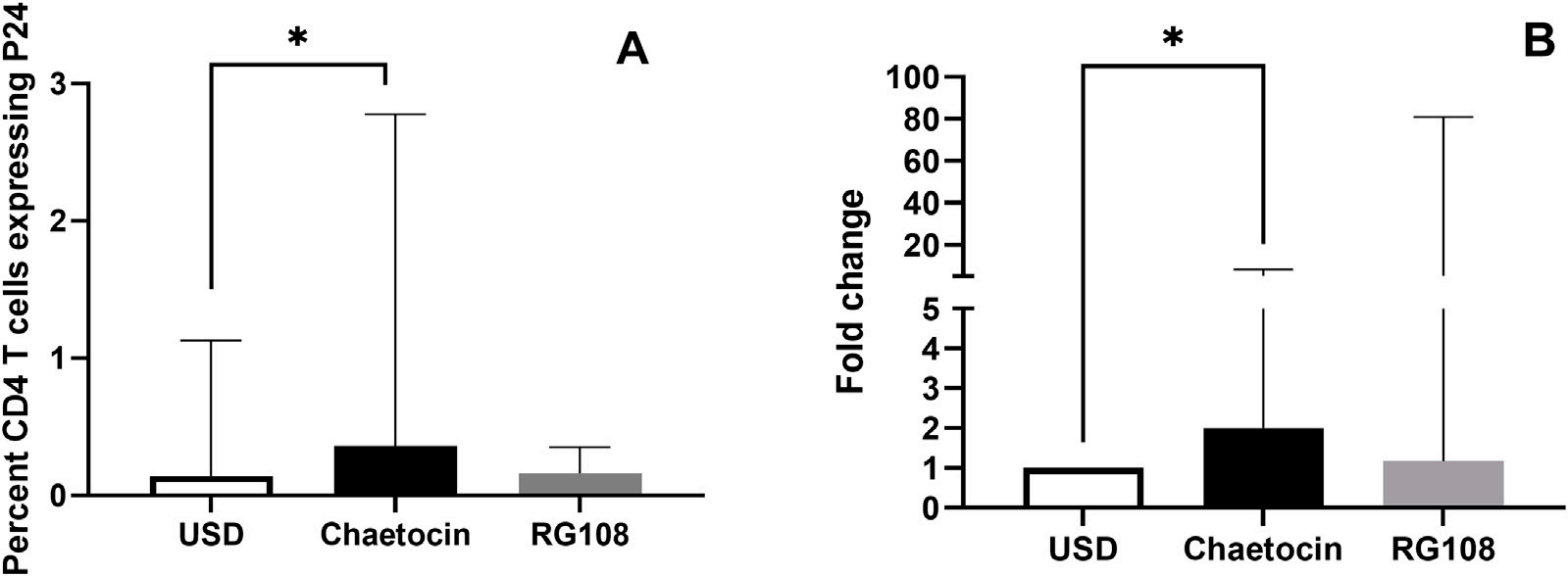
Effect of chaetocin treatment on prelamin A expression and HIV latency reversal in PLHIV cases: **(A)** intracellular P24 expression in CD4^+^ T cells of PLHIV with and without (USD) the treatment with chaetocin and RG108, as assessed by flow cytometry, is shown in the figure. **(B)** Fold-change of HIV-1 gag copies in the sorted CD4^+^ T cells of PLHIV with and without (USD) the treatment with chaetocin and RG108, as assessed by real-time PCR, is shown in the figure. Bars in the figure represent the medians, and error bars indicate the interquartile ranges for the values. P-values of the paired data were calculated using Wilcoxon signed-rank test.

The effect of chaetocin and RG108 treatment on HIV latency reversal is shown in Figure 7. HIV reactivation was assessed by intracellular P24 expression by flow cytometry and by estimating the fold change in HIV-1 gag copies in CD4^+^ T cells by real-time PCR. Chaetocin treatment was observed to result in significantly increased P24 expression and HIV-1 gag copies, indicating HIV reactivation (p = 0.0293 and 0.0268, respectively). Conversely, RG108 treatment did not show significant HIV reactivation in CD4^+^ T-cells.

## 4 Discussion

DNA damage plays a key role in aging processes, leading to alterations at the genetic, epigenetic, and structural levels (Schumacher et al., 2021). It can be caused by multiple endogenous and exogenous agents and occurs as the major consequence of oxidative stress. Failure of DNA damage response processes in maintaining DNA integrity results in the induction of cellular senescence or cell death. HIV infection is known to induce higher oxidative stress, even in PLHIV on ART. It also interferes in DNA damage response processes that are not restored completely after the initiation of antiretroviral therapy (Simenauer et al., 2020), increasing the risk of accelerated aging.

Aging has been connected to epigenomic alterations with genome-wide hypomethylation and promoter-specific hypermethylation that are common features of aging and age-related illnesses. DNA hypomethylation has been linked to frailty and loss of function, and it has been identified as a common hallmark of carcinogenesis. We assessed the levels of global methylation in whole-blood cells of PLHIV to determine if they demonstrate global hypomethylation. Cases enrolled in our study had significantly lower levels of global DNA methylation than the HIV-negative controls, indicating global hypomethylation. Lower global methylation levels have been reported in PLHIV than in the HIV-negative individuals (Zhang et al., 2016). There is a lack of evidence of Indian data on global methylation levels in PLHIV. Overexpression of DNMTs is shown to result in lower global methylation, and inhibitors of DNMTs have been shown to cause remodeling of global DNA hypomethylation in cancers (Liu et al., 2024). Histone methylation is also shown to facilitate DNA methylation by interacting with DNMTs (Li et al., 2021). HIV-1 infection has been shown to cause overexpression of HMTs along with DNMTs (Bogoi et al., 2018) (Mikovits et al., 1998), which might influence global methylation levels in PLHIV. Hence, we evaluated the effect of DNMT and HMT inhibitors such as RG108 and chaetocin on global methylation levels. The global methylation levels increased after treatment with these inhibitors, indicating their therapeutic potential in reversing global hypomethylation in PLHIV.

8-OHdG is a biomarker of oxidative DNA damage, which might result in somatic mutations acting as a driving force behind carcinogenesis (Valavanidis et al., 2009). 8-OHdG levels were significantly elevated in PLHIV in our study than in the HIV-negative participants. Higher 8-OHdG levels were also reported previously in PLHIV, which further increased after ART, indicating the role of ART in age acceleration in PLHIV (Kolgiri et al., 2017). We further observed a positive correlation of 8-OHdG levels with angiotensin-II levels. Angiotensin-II had been shown to increase oxidative stress, thereby increasing 8-OHdG levels (Uemura et al., 2008; Pialoux et al., 2017). Hence, angiotensin-II type-1 receptor blockers (ARBs) have been shown to exert a protective role in oxidative stress, as indicated by reduced 8-OHdG levels (Takao et al., 2009). Although 8-OHdG is a marker of oxidative DNA damage and linked with the aging process, it is also known to exert a regulatory feedback mechanism by acting as an antioxidant and anti-inflammatory agent (Ock et al., 2012). This might be the reason for the negative correlation observed between the levels of 8-OHdG and proinflammatory cytokines such as TNF-a and MCP-1 in our study. This could also be a reason for no significant difference in ROS levels between our cases and controls. 8-OHdG has been shown to exert inhibitory effects on macrophages and inhibit MCP-1 expression in vascular smooth muscle cells (Huh et al., 2012). Interestingly, we did not find any such negative correlation in HIV-negative controls. 8-OHdG was shown to exert the anti-inflammatory action in a dose-dependent manner (Kim et al., 2006), and since our controls had lower levels of 8-OHdG than the cases, we did not observe the negative correlation.

We also assessed prelamin A accumulation, which represents DNA damage at the structural levels. Oxidative stress acts as the cause and the effect of prelamin A accumulation (Ragnauth et al., 2010). PLHIV on a protease inhibitor (PI)-based regimen exhibited prelamin A accumulation due to the inhibition of ZMPSTE24 activity by PIs (Ragnauth et al., 2010). We observed higher prelamin A expression in PLHIV than in their age-matched controls in our study, although our study participants were not on a PI-based regimen. Studies determining prelamin A accumulation in PLHIV who are not on a PI-based regimen are lacking, and hence, the cause for higher prelamin A expression in such PLHIV needs to be investigated further. Percentages of CD57^+^ and CD25^+^ CD4^+^ T cells correlated positively with prelamin A accumulation, suggesting a link between immunosenescence, immune activation, and DNA damage. Studies on prelamin A accumulation in immunosenescent T cells are lacking and are required to be conducted to investigate the mechanisms interfering in DNA damage responses leading to immunosenescence. We further found a significant decrease in prelamin A expression after chaetocin treatment. Chaetocin treatment has been shown to extend the lifespan of progeria mice by sequestering prelamin A in the nuclear envelope and has been suggested as a treatment modality to slow down the progerin-induced aging process (Wang et al., 2024).

Epigenetic mechanisms also play an important role in the establishment and maintenance of HIV-1 latency. CpG methylation in the proviral promoter region regulates HIV maintenance in the latent state (Blazkova et al., 2009). In addition, studies using chromatin immunoprecipitation and inhibitors of epigenetic silencing complexes have shown that the major epigenetic restriction of the HIV provirus is due to histone methylation (Nguyen and Karn, 2024). Methylation at histone 3 lysine 27 (H3K27) and histone 3 lysine 9 (H3K9) has been shown to maintain HIV-1 latency in both primary and transformed T cells (Nguyen et al., 2017). Hence, inhibitors of DNMT and HMT have the potential of serving as HIV latency reversing agents. They have the ability to induce chromatin to a permissive state and stimulate viral transcription when administered as latency reversal agents (LRAs). While evaluating the effect of RG108 and cheatocin on HIV latency, we observed significant HIV reactivation only after chaetocin treatment. Chaetocin inhibits SUV39H1, thus reducing H3K9 trimethylation (H3K9me3), leading to chromatin relaxation. This globally alters gene expression, possibly upregulating the expressions of genes involved in prelamin A processing, such as ZMPSTE24, and genes involved in proteosomal degradation or autophagy that might help clear excess prelamin A. Chaetocin has been reported to reactivate HIV from CD4^+^ T cells isolated from HAART-treated PLHIV without inducing T cell response (Bouchat et al., 2012; Bernhard et al., 2011). It is also necessary to rule out the secretion of immunosuppressive cytokines by the cells after HIV reactivation, which might interfere in the elimination of the reservoir, as we have shown previously (Shete et al., 2016). Meanwhile, RG108 acts by blocking DNA methylation at CpG sites. Hence, it shows slower effects on global gene expression, especially on genes unrelated to direct CpG promotor methylation. Although there are no reports on HIV reactivation by RG108, other DNMTi such as 5-aza-2'deoxycytidine have been shown to mediate HIV reactivation in cell lines and HIV latency models using primary CD4^+^ T cells (Kauder et al., 2009). However, methylation of HIV DNA in the latently infected, resting CD4^+^ T cells of PLHIV on ART has been shown to be rare (Blazkova et al., 2012), and hence, we might not have observed a significant reactivation after RG108 treatment.

We performed this exploratory cross-sectional study in virally suppressed PLHIV to determine if they are at a risk for higher DNA damage potentially predisposing them to premature aging. However, a longitudinal study is required to confirm the associations between DNA damage markers and early aging. It would also be interesting to study these markers in PLHIV with virologic failure to determine the effect of uncontrolled viral replication on the markers of DNA damage response. Our sample size for the experiments with methylation modulators was limited as it was carried out with an exploratory objective to generate evidence, and more samples are required to be processed for confirming the results.

## 5 Conclusion

This study concluded that PLHIV were at a higher risk of DNA damage, as shown by global hypomethylation, higher 8-OHdG levels, and prelamin A-expressing T cells despite being on long-term ART, which might lead to genetic instability and increased susceptibility to accelerated immune aging. The association of global hypomethylation with immunosenescent and effector/effector memory T-cell phenotype indicated the role of global hypomethylation in altering the T-cell phenotype. A positive correlation between 8-OHdG and angiotensin-II levels suggested the possible role of angiotensin-II in mediating the oxidation of purines and, further, in DNA damage. A negative correlation between 8-OHdG and proinflammatory cytokine levels in PLHIV suggested a possible anti-inflammatory role of 8-OHdG in HIV infection. An increase in global methylation levels was observed after chaetocin treatment, which suggested its therapeutic potential in mitigating cellular aging in PLHIV. Reduction in prelamin A accumulation and simultaneous increase in P24-expressing CD4^+^ T-cells was observed after chaetocin treatment, which suggested its therapeutic potential in mitigating cellular aging in PLHIV and reversing viral latency.

## Data Availability

The original contributions presented in the study are included in the article/supplementary material further inquiries can be directed to the corresponding author.
